# Political Orientation as Psychological Defense or Basic Disposition? A Social Neuroscience Examination

**DOI:** 10.3758/s13415-021-00965-y

**Published:** 2021-11-11

**Authors:** Kyle Nash, Josh Leota

**Affiliations:** 1 Department of Psychology, University of Alberta, Edmonton, AB Canada; 2 Present Address: Turner Institute for Brain and Mental Health, School of Psychological Sciences, Monash University, Melbourne, VIC 3168 Australia

**Keywords:** Political orientation, EEG, ERP, P3, Negativity bias

## Abstract

Psychological views on political orientation generally agree that conservatism is associated with negativity bias but disagree on the form of that association. Some view conservatism as a *psychological defense* that insulates from negative stimuli and events. Others view conservatism as a consequence of increased *dispositional sensitivity* to negative stimuli and events. Further complicating matters, research shows that conservatives are sometimes more and sometimes less sensitive to negative stimuli and events. The current research integrates these opposing views and results. We reasoned that conservatives should typically be less sensitive to negative stimuli if conservative beliefs act as a psychological defense. However, when core components of conservative beliefs are threatened, the psychological defense may fall, and conservatives may show heightened sensitivity to negative stimuli. In two ERP studies, participants were randomly assigned to either an ostensibly real economic threat or a nonthreatening control condition. To measure reactivity to negative stimuli, we indexed the P3 component to aversive white noise bursts in an auditory oddball paradigm. In both studies, the relationship between increased conservatism and P3 mean amplitude was negative in the control condition but positive in threat condition (this relationship was stronger in Study 2). In Study 2, source localization of the P3 component revealed that, after threat, conservatism was associated with increased activity in the anterior cingulate cortex and dorsomedial prefrontal cortex, regions associated with conflict-related processes. These results demonstrate that the link between conservatism and negativity bias is context-dependent, i.e., dependent on threat experiences.

## Introduction

Psychological theories of political orientation can be organized into two types, both focused on the mechanisms underlying conservative belief; 1) *Conservatism as psychological defense*: characterized by clarity, certainty, and resistance to change; conservative ideology embeds the individual in a stable psychological system that can compensate for heightened vulnerability or sensitivity to negativity, threat, and/or ambiguity (e.g., motivated social cognition, Jost et al., 2003); 2) *Conservatism as dispositional sensitivity*: characterized by beliefs that promote security, tradition, hierarchy, and structure; conservative ideology is a cold cognitive consequence of dispositional sensitivity to negative stimuli and events (e.g., negativity bias, Hibbing et al., 2014).

These two theories essentially agree that conservatives are linked to some kind of heightened vulnerability or sensitivity to aversive or negative stimuli (perspectives vary on the precise nature of that sensitivity, e.g., is this sensitivity to stimuli that induce disgust, threat, fear, cognitive inconsistency, arousal, etc., see the target article and responses to Hibbing et al., 2014). However, the psychological defense view holds that conservatism places the individual in a “black and white” psychological system that limits ambiguity, promotes social consensus, and prescribes clear guides for action (Jost & Amodio, 2012; Jost et al., 2007). This belief system thus compensates for and mutes that initial vulnerability and heightened sensitivity to negativity. The dispositional sensitivity view holds that conservative beliefs are more of an offshoot of the individual, stable differences in neurocognitive functioning, i.e., a protective and security-based ideology that directly results from increased sensitivity to potential threats and conflicts. In sum, though the origins of political ideology are consistent across these two views (i.e., a negativity bias), they diverge on the functional consequences of negativity bias. That is, the psychological defense view would suggest that, compared with liberals, conservatives would be protected against or *less* responsive to negative stimuli under normal (i.e., nonthreatening) circumstances. The dispositional view would suggest that, compared to liberals, conservatives would be vulnerable or *more* responsive to negative stimuli under normal circumstances. Empirical evidence is similarly divided (Smith & Warren, 2020).

Consistent with the psychological defense view, conservatives have been found to be *less* sensitive to negative stimuli and events. For example, in event-related potential (ERP) research, political orientation has been associated with error-related negativity (ERN, Amodio et al., 2007; Weissflog et al., 2013), an ERP component elicited by the negative event of committing a response error (Hajcak & Foti, 2008). Specifically, conservatives, compared to liberals, demonstrated reduced ERN amplitudes to error commission in a reaction time task, suggesting that they are less sensitive to conflict or aversive events. Conservatism and moral beliefs associated with conservatism have both been linked to decreased cortical volume in the anterior cingulate cortex (ACC; Kanai et al., 2011; Nash et al., 2017), a brain area important in conflict detection, negative affect, and pain (Shackman et al., 2011). Poignant threats, such as thoughts of death, encounters with pathogens, terrorist attacks, and economic troubles, have been found to increase conservative social cognition and heighten support for conservative beliefs and values (Bonanno & Jost, 2006; Echebarria-Echabe & Fernández-Guede, 2006; Nail et al., 2009; Ullrich & Cohrs, 2007; McGregor et al., 2001; Sales, 1972; Van Leeuwen et al., 2012; Van de Vyver et al., 2016; Wright & Baril, 2013). Finally, conservatives have been found to be more emotionally stable, or less neurotic (Gerber et al., 2010).

Consistent with the dispositional sensitivity view, conservatives have been found to be *more* sensitive to negative stimuli and events. For example, conservatives demonstrate increased skin conductance, indicating increased sympathetic arousal, to aversive images, including a large spider or an open wound (Dodd et al., 2012). Similarly, conservatives demonstrate increased corrugator muscle activation, indicating increased negative affect, to a negative social scenario (Fodor et al., 2008), and increased blink amplitude, indicating a heightened fear state, to sudden auditory stimuli (Oxley et al., 2008). Economic conservatism is associated with increased neural connectivity between the bed nucleus of the stria terminalis (BNST) and the amygdala during threat versus safety, a neural circuit associated with responding to sustained or uncertain threats (Pedersen et al., 2018). Conservatives are more cautious in the exploration of novel stimuli (Shook & Fazio, 2009). A meta-analysis reveals that conservatives prefer cognitive closure, dislike ambiguity, and experience higher levels of death anxiety (Jost et al., 2003).

These contrasting findings invite obvious questions. If conservatism is a type of psychological defense against negativity, then why are conservatives sometimes more reactive and vulnerable to a variety of basic, negative events? If conservatism is grounded in dispositional negativity bias, then why are conservatives sometimes less sensitive to negative events? Unfortunately, this area of research has been further complicated by the fact that the same effects have been put forward in support of both views (in these interpretations, directionality of the effect often is ignored).

We conducted two electroencephalographic (EEG) studies that could potentially integrate these contradictory views of political orientation. In both studies, we first measured political orientation and then randomly assigned participants to experience either a poignant and ostensibly real threat to the economic system in an effort to compromise the ability of conservative beliefs to act as a defense or a nonthreatening control condition. Conservatives are characterized by a motivation to protect existing societal institutions, such as the current economic system (Janoff-Bulman, 2009) and are more likely to embrace economic system-justifying ideologies, including meritocratic, just-world, and fair market ideologies (Jost & Hunyady, 2005). We reasoned that a legitimate threat to the individual’s place in the economic system—essentially signaling that the current economic institutions are unfair, uncertain, and flawed—would jeopardize core motivational underpinnings of conservatism. Prior research shows that direct threats to psychological defenses increase sensitivity to negativity (Gyurak & Ayduk, 2007; Holbrook et al., 2011; Nash et al., 2014; Schimel et al., 2007). If conservatism is indeed a psychological defense that confers stability and certainty, then that defense would be less effective in the face of economic instability and uncertainty. We then measured basic neurocognitive processes in encountering negative stimuli—white noise bursts in an auditory oddball paradigm. In Study 1, we examined ERPs using a 14-channel mobile EEG system. In Study 2, we used a 64-channel EEG system that enabled source localization analyses (sLORETA; Pascual-Marqui, 2002). Study 1 was conducted in New Zealand and Study 2 was conducted in Canada. We expected that the link between conservatism and negativity bias is context-dependent (for a related account on context, ideology, and emotion, see Pliskin et al., 2020). Specifically, we predicted that, as a compensatory and protective ideology, conservatism would be associated with reduced reactivity to negative stimuli in the control condition, a non-threatening context that is closest to a neutral or baseline state. However, upon experiencing a threat to the psychological foundations of that protective ideology, conservatism would be associated with increased reactivity to negative stimuli.

To index reactivity to aversive stimuli, we used the white noise P3 in a passive auditory oddball task. The P3 is a large, positive ERP component commonly elicited by aversive, startling, or novel cues (i.e., “oddball” stimuli; Sutton et al., 1965). According to the locus coeruleus-norepinephrine (LC-NE) hypothesis of the P3 (Nieuwenhuis et al., 2005), the P3 reflects activation of the LC-NE system and is the electrophysiological analogue of emotional arousal (Nieuwenhuis et al., 2005; see also adaptive gain theory; Servan-Schreiber et al., 1990; Aston-Jones & Cohen, 2005). The link between negative arousal and P3 amplitude is most prominent in passive auditory oddball tasks, at frontal electrodes, and in response to aversive or infrequent stimuli (Combs & Polich, 2006; Ermutlu et al., 2005; Grillon & Ameli, 1994; Nieuwenhuis et al., 2005). Therefore, we tested if Economic Threat increased P3 mean amplitude to random and infrequent white noise bursts at frontal electrodes among liberals and conservatives.

## Study 1

### Method

#### Participants and design

Ethical approval for this study was obtained from the University of Canterbury Human Ethics Committee. Pilot data indicated that the current economic threat manipulation had a medium to large effect size on self-reported anxiety (Cohen’s *d* = 0.65). Therefore, we aimed to include 30 individuals per condition (power analyses in G*Power: difference between two independent groups, *expected effect size* Cohen’s *d* = 0.65, *power* = 0.80, *alpha* = 0.05, and *number of groups* = 2, *one-tailed, output sample size* = 60). Participants (*N* = 60; *M*_age_ = 20.18 years; females = 46) were selected from a New Zealand undergraduate psychology class and were compensated with class credit. The study used a between-subjects design with random assignment into two conditions (*Economic Threat* vs. *No-Threat* Control). Political Orientation was included as a moderator variable and reactivity to negative stimuli, measured as white noise P3 mean amplitude within a passive auditory oddball paradigm, served as the dependent variable.

#### Procedure

Participants were seated at individual computers within a lab (up to 2 participants at a time), provided written, informed consent, and then were fitted with a 14-electrode, mobile EEG headset (Emotiv EPOC + , Emotiv Systems Inc., San Francisco, CA). Materials were completed on a desktop computer using Qualtrics and ePrime software. Participants first completed demographic and personality questionnaires to strengthen our cover story that we were interested in how different personality variables correlated with one another (all data available upon request), and were then randomly assigned to either the Economic Threat condition or the No-Threat Control condition. Participants then completed the auditory oddball paradigm. Finally, participants completed a retrospective measure of self-reported affect to the economic threat (vs. control) manipulation. Participants were then thoroughly debriefed and thanked for their efforts.

#### Political Orientation

A single-item measure of Political Orientation was recorded on a 7-point scale from *very liberal* to *very conservative* with *moderate or center* as a midpoint. This single-item measure is the most widely used measure of political ideology in political psychological research (Sibley et al., 2012), exhibits high predictive validity (Jost, 2006; Jost et al., 2009), test–retest reliability (Knight, 1999), and is widely used in past political neuroscience research (Amodio et al., 2007; Kanai et al., 2011). In the present study, the responses were normally distributed (*M* = 3.530; *SD* = 0.947). The full continuous measure was used in all statistical analyses.

#### Economic Threat Manipulation

Participants were tasked with creating a headline for an ostensibly real newspaper article that they were told had appeared that month in *The Press*, a local newspaper in New Zealand. In the Economic Threat condition, participants read an article detailing a very pessimistic economic future in New Zealand. This forecast was compiled by leading New Zealand economists. Critically, the article suggested that young adults would be hit hardest. Therefore, this manipulation was tailored to elicit economic angst among our sample (undergraduate students). In the No-Threat Control condition, participants read a similar article detailing a more stable economic future in New Zealand. Both articles were constructed from real, publicly available marcoeconomic forecasts from financial media outlets. Participants were asked to submit their headline after the auditory oddball paradigm and before the manipulation check.

#### Auditory Startle Paradigm

Participants listened passively on headphones to a series of frequent standard tones (pure 1,000 Hz tones for 50 ms, headphone volume setting 50 in Windows) and less frequent white noise blasts (2:8 white noise to standard). Each stimulus was presented for a second and the entire paradigm lasted for three minutes, for a total of 180 trials (approximately 36 white noise and 144 beep trials). To minimize movement, participants were asked to fixate on a small cross presented on the computer screen during the task.

#### EEG Recording and Preprocessing

During the auditory startle task, EEG was recorded using a 14-channel (gold-plated contact-grade hardened copper with felt pads moistened with saline) Emotiv EEG wireless headset (Emotiv Systems Inc., San Francisco, CA), and Emotiv TestBench software at a sampling rate of 128 Hz. The 14 channels, AF3, AF4, F3, F4, F7, F8, FC5, FC6, P7, T7, T8, P8, O1, and O2, were positioned according to the 10–20 International System. The left mastoid electrode was used as online reference. The Emotiv headset system has proved a reliable, quick-application alternative to standard systems in measuring ERPs to auditory oddball stimuli, including indexing the P3 components (Badcock et al., 2013; Mayaud et al., 2013). Emotiv EEG technology has become an increasingly popular alternative to standard EEG systems in social and cognitive neuroscience research (Agroskin et al., 2016; Louwerse & Hutchinson, 2012; Steinhubl et al., 2015) and in brain-computer interface (BCI) applications (Bobrov et al., 2011; Choi & Jo, 2013; Debener et al., 2012; De Vos et al., 2014; Khushaba et al., 2013; O’Regan & Marnane, 2013; Vourvopoulos & Liarokapis, 2014).

Using the analysis software Brain Vision Analyzer 2.0 (BVA 2), EEG data were re-referenced to the average mastoids and band-pass filtered between 0.1 and 30 Hz. Blinks were statistically removed using the automatic ocular independent component analysis (HEOG and VEOG reference electrode = AF3) in BVA 2 which isolates and deletes blink related factors. Artefacts were detected using the following parameters: − 100 to + 100 μV min/max threshold, 50 μV maximum voltage step, 0.5 μV lowest allowed voltage (max – min) in 100-ms intervals. Recordings were then segmented into 1,000-ms epochs locked on either standard or white noise presentation, 200 ms before–800 ms after the stimulus. All artefact free epochs were then averaged, creating average ERPs of standard and white noise tones for each participant. Each average ERP was baseline-corrected by subtracting the average voltage during the 200–0 ms time period before stimulus presentation. As in previous research using the Emotiv EEG wireless system with a Bluetooth connection (Nash et al., 2019) and research examining the link between negative arousal and P3 amplitude (Combs & Polich, 2006; Ermutlu et al., 2005; Grillon & Ameli, 1994; Nieuwenhuis et al., 2005), the P3 was quantified for both standard tone and white noise stimuli as the mean amplitude between 275 to 475 ms after stimulus presentation at the frontocentral site where the startle component was maximal, the electrode F4.

#### Manipulation Check

Before debriefing, participants were asked to retrospectively rate how “*reading The Press News article*” made them feel on a range of positive and negative affective labels (these included Good, Happy, Smart, Successful, Likeable, Meaningful, Frustrated, Confused, Uncertain, Empty, Anxious, Ashamed, Insecure, Lonely, Stupid, Out of Control, and Angry; McGregor et al., 2010a; b; Nash et al., 2014; Schumann et al., 2014). We computed a Felt-anxiety-composite (Cronbach’s α = 0.833) as a self-reported measure of anxiety from all anxiety-related adjectives (including Anxious, Uncertain, and Frustrated, see Nash et al., 2011, for evidence that a theoretically-specific anxiety manipulation causes an increase in these three items).

#### Statistical Analyses

We first examined if political orientation moderated the effect of the economic threat manipulation on P3 mean amplitudes. We conducted moderated multiple regression using the Process Macro in SPSS (Model 1; Hayes, 2017), with the condition variable entered as the grouping variable, political orientation as a continuous moderator variable, and P3 mean amplitude at F4 as the dependent variable. To test the hypothesis that the relationship between conservatism and negativity bias is context-dependent, we performed both simple slope analyses and simple effect analyses. First, simple slope analyses allowed us to test whether conservatives, compared to liberals, were less reactive to negative stimuli in the No-Threat Control condition (psychological defense view) and more reactive to negative stimuli in the Economic Threat condition (dispositional sensitivity view). Second, simple effect analyses allowed us to test whether Economic Threat specifically caused conservatives to become more reactive to negative stimuli, thus supporting the idea that a threat to the conservative worldview may cause the psychological defenses to fall, leaving conservatives vulnerable.

To remove processes common to both stimuli, we examined the P3 component calculated from the difference wave between white noise and standard tones. We conducted the same moderated multiple regression analysis with P3 mean amplitude from the white noise difference wave entered.

### Results

#### Manipulation check

A one-way ANOVA demonstrated that participants in the Economic Threat condition reported higher Felt-anxiety-composite scores (*M* = 3.353, *SD* = 0.734) than participants in the No-Threat Control condition (*M* = 2.309, *SD* = 0.773), *F* (1, 59) = 29.075, *p* < 0.001, η^2^_*p*_ = 0.33. Thus, the Economic Threat manipulation caused greater self-reported anxiety in our sample, compared to the No-Threat Control task.

#### Auditory Oddball Task

**Fig. 1:**
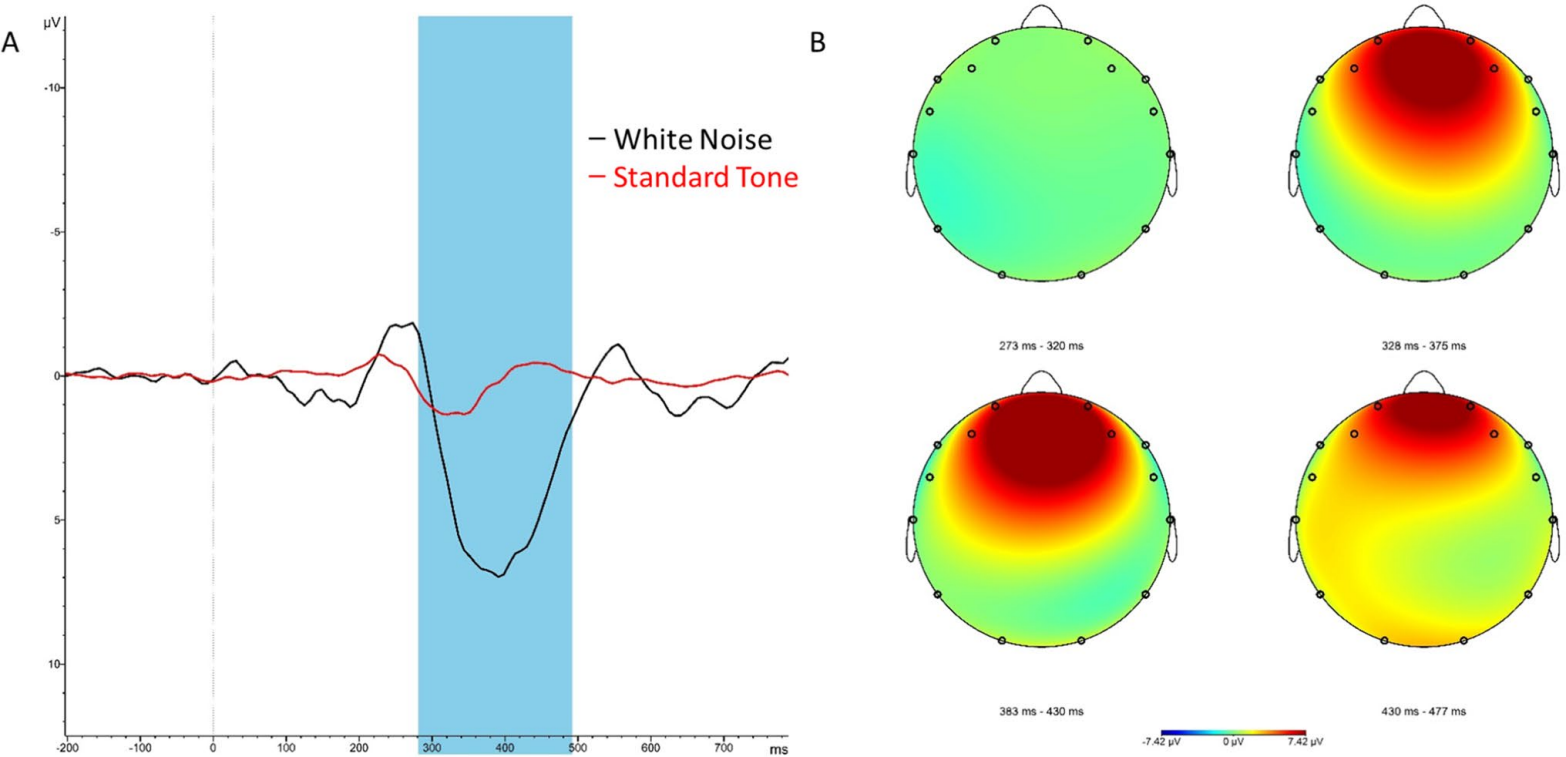
**A.**Study 1 grand averaged event-related potentials to white noise (black) and standard tones (red) at electrode F4. The P3 mean amplitude was calculated from 275–475 ms (highlighted). **B.** Grand averaged P3 topographies to white noise stimuli in four separate windows of the P3, demonstrating maximal activation at F4 (at 391 ms)

**Fig. 2:**
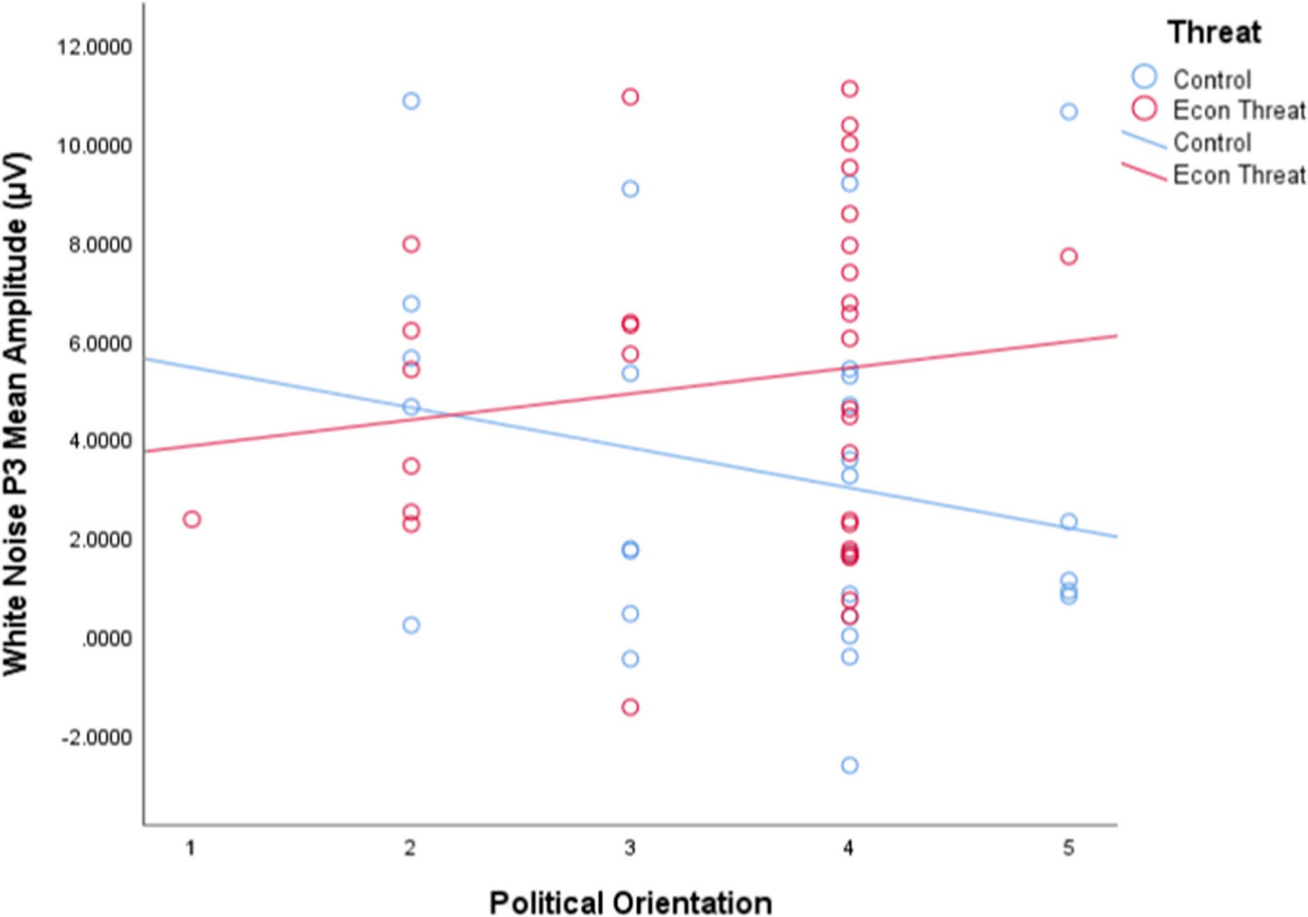
Study 1 scatterplot of the interaction between condition and political ideology on P3 mean amplitude to white noise (275–475 ms) at electrode F4

We first conducted a moderated multiple regression analysis of the impact of condition, political orientation, and their interaction on white noise P3 mean amplitudes (Fig. 1). Results revealed a non-significant condition by political orientation interaction effect, *t*(56) = 1.528, *p* = 0.132 (Fig. 2). Simple slope analyses revealed that in the control condition, there was a nonsignificant but negative relationship between increased conservatism and white noise P3 mean amplitudes, *t*(56) =  − 1.318, *p* = 0.193. In the Economic Threat condition, there was a nonsignificant but positive relationship between increased conservatism and white noise P3 mean amplitudes, *t*(56) = 0.835, *p* = 0.407. However, an examination of the conditional effect at conservative orientation (defined as + 1 SD on political orientation) revealed that conservatives showed significantly higher white noise P3 mean amplitudes in the Economic Threat condition (*M* = 5.684), compared with conservatives in the control condition (*M* = 2.454), *t*(56) = 2.612, *p* = 0.012. There was no conditional effect at liberal orientation (defined as − 1 SD on political orientation, *p* = 0.667).

Results using the difference score for P3 mean amplitudes between white noise and standard tone trials revealed a nonsignificant interaction effect, *t*(56) = 1.288, *p* = 0.203. Simple slope analyses revealed that in the control condition, there was a nonsignificant negative relationship between increased conservatism and difference wave P3 mean amplitudes, *t*(56) =  − 1.474, *p* = 0.146. In the Economic Threat condition, there was a nonsignificant positive relationship between increased conservatism and difference wave P3 mean amplitudes, *t*(56) = 0.323, *p* = 0.746. However, an examination of the conditional effect at conservative orientation (+ 1 SD) showed significantly higher difference wave P3 mean amplitudes in the Economic Threat condition (*M* = 4.908), compared with conservatives in the control condition (*M* = 2.329), *t*(56) = 2.157, *p* = 0.035. There was no conditional effect at liberal orientation (− 1 SD, *p* = 0.736).^1^ Together, these results show that Economic Threat increased sensitivity to negative stimuli (white noise blasts) for conservatives, but not for liberals.

## Study 2

Study 1 demonstrated preliminary support for the psychological defense view of conservatism. Results showed that upon experiencing a poignant economic threat, conservatism was associated with increased reactivity to aversive stimuli. Although nonsignificant, in the control condition, conservatives appeared less sensitive than liberals. This suggests that conservatism does indeed act as a kind of psychological defense against aversive experiences, that is, until the defense itself (a stable and certain system and worldview) is threatened (by economic angst), and conservatives are revealed as more sensitive to the same aversive experiences. Though conservatives appeared less sensitive to liberals in the control condition and vice versa in the threat condition, in contrast to the conditional effect amongst conservatives, the results of the simple slope analyses failed to reach significance. Therefore, the results from Study 1 are not robust enough—alone—to refute the null hypothesis. To follow-up on these suggestive results, we ran a second study with a larger sample size and an EEG system with increased spatial resolution.

Study 2 thus replicated and extended Study 1. Note that Study 2 is a partial re-analysis of data published in Nash et al. (2020). In this study, participants were randomly assigned to highly similar economic threat conditions (adapted to apply to the population) and then completed the same auditory oddball task. EEG was recorded using a 64-channel system that allowed source localization analyses (sLORETA; Pascual-Marqui, 2002) to better characterize the impact of economic threat and political ideology on the neural mechanisms underlying the P3.

### Method

#### Participants

Ethical approval for this study was obtained from the University of Alberta Human Research Ethics Board (Protocol 00084513). Participants (*N* = 110; mean age = 19.78; age range = 17–26 years; 61 females) with normal or corrected-to-normal vision were recruited from a first-year introductory psychology class and earned class credit. Study 1 demonstrated that the current manipulation had a medium-to-large effect size on conservatives and P3 amplitude (Cohen’s *d* = 0.623). We sought 50 individuals per condition and collected data until the end of the 2019 fall term (power analyses in G*Power: difference between two independent groups, *d* = 0.623, *alpha* = 0.05, *power* = 0.80, and *number of groups* = 2, *total sample size* = 66). A total of ten participants were excluded due to poor connectivity (*N* = 6), missing EEG data (*N* = 2), or an extreme P3 mean amplitude (*N* = 1, *Z score* = 3.95, all other Z-scores <  ± 2.5), leaving 100 participants for analyses.

#### Procedure

Participants were seated at a computer station in an electrically- and sound-shielded room. They first completed an informed consent and were then fitted with a 64-channel EEG headset (Brain Products). Participants then answered demographic questions and several personality questionnaires as part of a larger research project on individual differences in the neuroscience of self-regulation (all data available upon request). This section included a measure of political orientation (see below). Participants were then randomly assigned to either the *Economic Threat* condition or the *No-Threat Control* condition. Participants then completed the passive auditory oddball task, the primary task used here. Again, as part of a separate line of research, participants then completed a color-naming Stroop task, and a Balloon Analogue Risk-Taking task (manuscripts in prep.). After, participants completed a wealth justification scale. Finally, participants completed manipulation and compliance checks. Participants were then debriefed, had the headset removed and hair washed, and thanked for their time.

#### Political Orientation

Responses to a single-item measure of political orientation were recorded using the same 7-point scale used in Study 1, from *strongly liberal* to *strongly conservative* with *moderate or center* as a midpoint. In the present study, the responses were normally distributed (M = 3.550, *SD* = 1.232).

#### Economic Threat Manipulation

The Economic Threat manipulation was based off the manipulation used in Study 1. Participants in the Economic Threat condition read an ostensibly real online article from CBC.ca about a troubling economic forecast in Canada that would have the largest impact on young adults. The forecast was putatively compiled by top Canadian researchers who concluded that a recession was imminent and that students would be hit hardest given the vulnerable position they were left in by the 2008 economic crisis. As such, this article was tailored to participants in our sample, i.e., young students. Participants in the No-Threat Control condition read an ostensibly real article from CBC.ca about a more neutral economic forecast that emphasized stability and a continuation of the status quo. Notably, both forecasts were based on real, publicly available economic predictions from financial news outlets.

#### Passive Auditory Oddball Paradigm

Immediately after the economic threat manipulation, participants listened to a series of standard tones (pure 1,000 Hz tones for 50 ms) and white noise bursts (0–20,000 Hz “hissing” sound for 50 ms, both at volume setting 50 in Windows) presented at 75 dB SPL and 80 dB SPL, respectively. The ratio of white noise to standard tones was 1:9. A stimulus was presented each second and the entire paradigm lasted for 3.5 min, for a total of 210 trials (approximately 21 white noise and 189 standard tone trials). Participants were informed that they would hear noises but were not instructed to do anything other than fixate on a small circle presented on the computer screen during the task.

#### EEG Recording and Preprocessing

Continuous EEG was recorded using the 64 Ag–AgCl channel ActiCHamp EEG system (Brain Products), positioned according to the 10/10 system and digitized at a sampling rate of 512 Hz (24 bit precision; bandwidth: 0.1–100 Hz). During recording, signals were referenced to TP9 electrode positioned over the left mastoid. Offline, EEG was re-referenced to the average mastoids (TP9-TP10), down-sampled to 256 Hz, band-pass filtered between 0.1 and 30 Hz, and notch filtered at 60 Hz. Blinks were statistically removed using the automatic ocular correction developed by Gratton et al. (1983). Artifacts were then automatically detected using the following parameters: − 100 to + 100 μV min/max threshold, 50 μV maximum voltage step, 0.5 μV lowest allowed voltage (maximum–minimum) in 100-ms intervals. Data were segmented into 1,000-ms epochs locked on either standard tone or white noise presentation, 200 ms before to 800 ms after the stimulus. For each participant, all artifact-free epochs were then baseline-corrected by subtracting the average voltage during the -200–0 ms time period before the stimulus and averaged, creating average ERPs of standard tone (average per participant = 184.29) and white noise (average per participant = 20.58). Individual P3 mean amplitudes were calculated for both standard tones and white noise stimuli as the mean amplitude between 200 and 400 ms after stimulus, at the frontocentral site where the startle component was maximal, the electrode FCz.

#### Source Localization of the P3

Standardized low-resolution electromagnetic tomography (sLORETA; Pascual-Marqui, 2002) was used to estimate the intracerebral electrical sources that generated the scalp-recorded activity during auditory oddball stimuli. The sLORETA method is a standardized, discrete, 3D distributed, linear, minimum norm inverse solution that allows for localization of the intracerebral sources of scalp-recorded electromagnetic signals. sLORETA has been validated in several simultaneous EEG/fMRI studies (Mobascher et al., 2009a, b) and in an intracortical EEG localization study for epilepsy (Rullmann et al. 2009). In the current implementation of sLORETA, computations are conducted in a realistic head model using the MNI152 template (Mazziotta et al. 2001), with the 3D solution space restricted to cortical gray matter, as determined by the probabilistic Talairach atlas (Lancaster et al. 2000). The intracerebral volume is partitioned into 6239 voxels at 5-mm spatial resolution. sLORETA images represent the standardized electric activity at each voxel in neuroanatomic Montreal Neurological Institute (MNI) space as the magnitude of estimated current density. For each participant, sLORETA images were computed for scalp-recorded activity for both the white noise and standard tone average ERPs. These images were normalized to a total current density of one and log-transformed.

#### Manipulation check

As in Study 1, participants were asked to retrospectively rate how “*reading the CBC News article*” made them feel. The same Felt-anxiety-composite (Cronbach’s α = 0.833) as Study 1 was created as a self-reported measure of anxiety from all anxiety-related adjectives (including anxious, uncertain, and frustrated).

#### Statistical Analyses

We first examined if political orientation moderated the effect of the economic threat manipulation on P3 mean amplitudes. We conducted moderated multiple regression using the Process Macro in SPSS (Model 1, see Hayes, 2017), with the condition variable entered as the grouping variable, political orientation as a continuous moderator variable, and P3 mean amplitude at FCz as the dependent variable. As in Study 1, we performed both simple slope analyses and simple effect analyses and also examined the difference score for P3 mean amplitudes between white noise and standard tone trials at FCz to remove processes common to both stimuli.

**Fig. 3:**
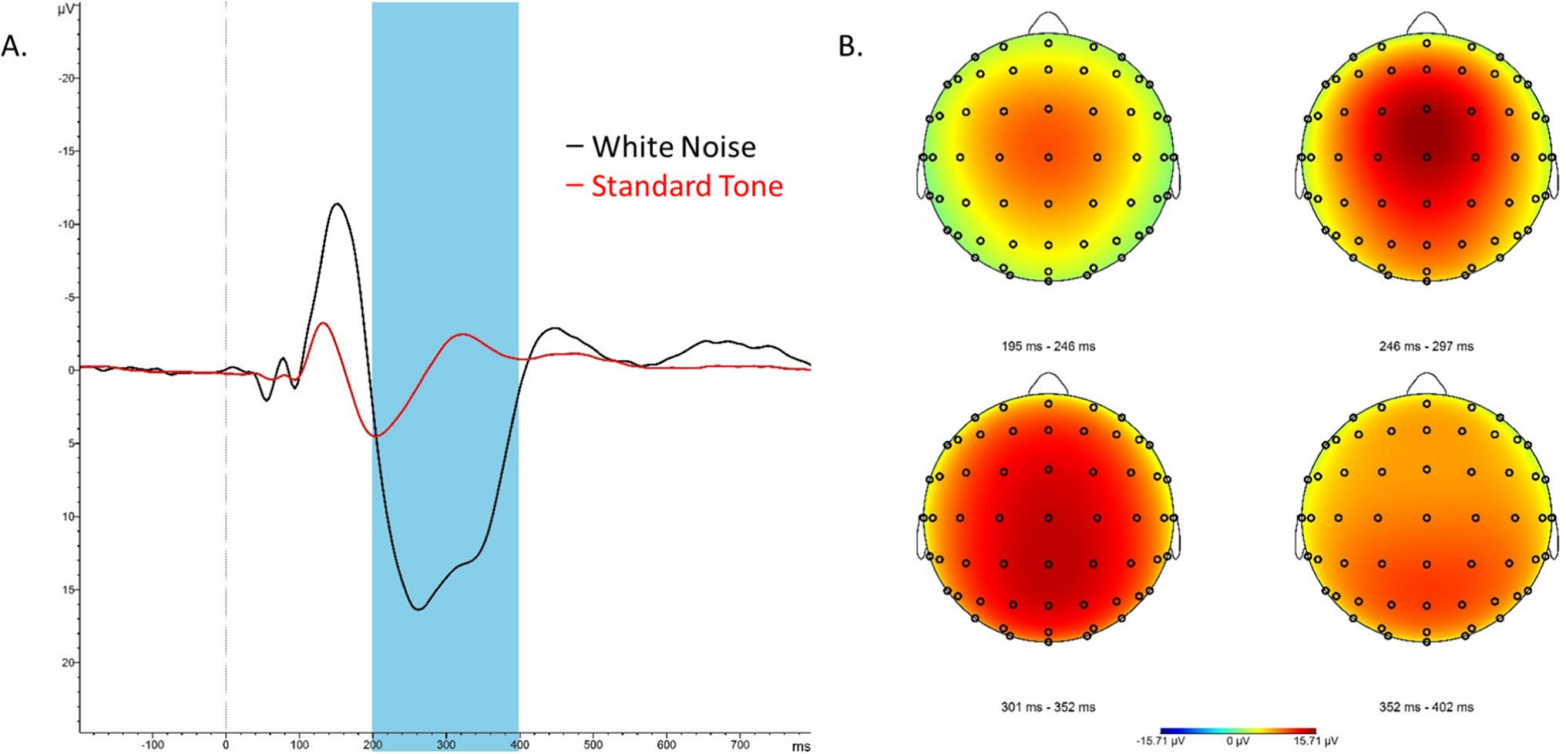
**A.**Study 2 grand averaged event-related potentials to white noise (black) and standard tones (red) at electrode FCz. The P3 mean amplitude was calculated from 200–400 ms (highlighted). **B.** Grand averaged P3 topographies to white noise stimuli in four separate windows of the P3, demonstrating maximal activation at FCz (at 264 ms)

We next examined if political orientation moderated the effect of the economic threat manipulation on the paired contrast between white noise and standard tone sLORETA images, specifically at the P3 timeframe. The standard tone sLORETA images were subtracted from the white noise sLORETA images during analyses to remove processes common to both stimuli (as in Study 1), allowing more isolated focus on conflict and arousal to white noise. In MATLAB, whole-brain voxel-by-voxel moderated multiple regression tests of the sLORETA images were conducted on the timeframes during the P3 component (200–400 ms; Fig. 3), with the condition variable and political orientation entered as first-level predictors, and their interaction term entered as a second-level predictor (MATLAB script available upon request). Correction for multiple testing for all 6239 voxels was implemented by means of a nonparametric randomization approach (Nichols & Holmes, 2002). This approach estimates empirical probability distributions and the corresponding critical probability thresholds (corrected for multiple comparisons). We expected that the economic threat manipulation would cause increased activation in the dorsal ACC during the P3 timeframe, particularly amongst those oriented towards conservatism, indicating heightened sensitivity to negative stimuli.

### Results

#### Manipulation Check

A one-way ANOVA demonstrated that participants in the Economic Threat condition reported higher Felt-anxiety-composite scores (*M* = 3.774, *SD* = 0.872) than participants in the No-Threat Control condition (*M* = 2.265, *SD* = 0.939), *F* (1,101) = 70.672, *p* < 0.001, η^2^_*p*_ = 0.412. Thus, the Economic Threat manipulation caused greater self-reported anxiety in our sample, compared with the No-Threat Control task.

#### Auditory Oddball Task

As shown in Fig. 3, P3 topographies demonstrated a frontocentral positivity, maximal at the FCz electrode. Notably, there were similar peak latencies after white noise stimulus presentation across conditions (P3: Economic Threat = 262 ms; No-Threat Control = 266 ms).

We first conducted a moderated multiple regression analysis of the impact of condition, political orientation and their interaction on white noise P3 mean amplitudes. One outlier was excluded from these analyses (see Participants section). Results revealed a significant condition by political orientation interaction, *t*(96) = 2.901, *p* = 0.005. Simple slope analyses revealed that in the control condition, there was a significant negative relationship between increased conservatism and white noise P3 mean amplitudes, *t*(96) =  − 2.003, *p* = 0.048. In the Economic Threat condition, there was a significant positive relationship between increased conservatism and white noise P3 mean amplitudes, *t*(96) = 2.165, *p* = 0.033. An examination of the conditional effects of condition for liberal orientation (defined as − 1 SD on political orientation) and conservative orientation (defined as + 1 SD on political orientation) revealed that liberals showed significantly reduced white noise P3 mean amplitude in the Economic Threat condition (M = 9.146), compared with liberals in the No-Threat control condition (M = 13.769), *t*(96) =  − 2.507, *p* = 0.014. In contrast, conservatives showed significantly higher white noise P3 mean amplitude in the Economic Threat condition (M = 12.888), compared to conservatives in the control condition (M = 9.156), *t*(96) = 1.999, *p* = 0.048.

Results using the difference score for P3 mean amplitudes between white noise and standard tone trials also revealed a significant condition by political orientation interaction, *t*(96) = 2.634, *p* = 0.01. Simple slope analyses revealed that in the control condition, there was a marginally significant negative relationship between increased conservatism and white noise P3 mean amplitudes, *t*(96) =  − 1.946, *p* = 0.055. In the Economic Threat condition, there was a marginally significant positive relationship between increased conservatism and white noise P3 mean amplitudes, *t*(96) = 1.793, *p* = 0.076. Examination of the conditional effects revealed that liberals again showed significantly reduced white noise P3 mean amplitude difference score in the Economic Threat condition (M = 9.453) compared to liberals in the No-Threat control condition (M = 13.713), *t*(96) =  − 2.348, *p* = 0.021. In contrast, conservatives showed marginally higher P3 mean amplitude difference score in the Economic Threat condition (M = 12.486), compared with the control condition (M = 9.330), *t*(96) = 1.741, *p* = 0.085.

**Fig. 4:**
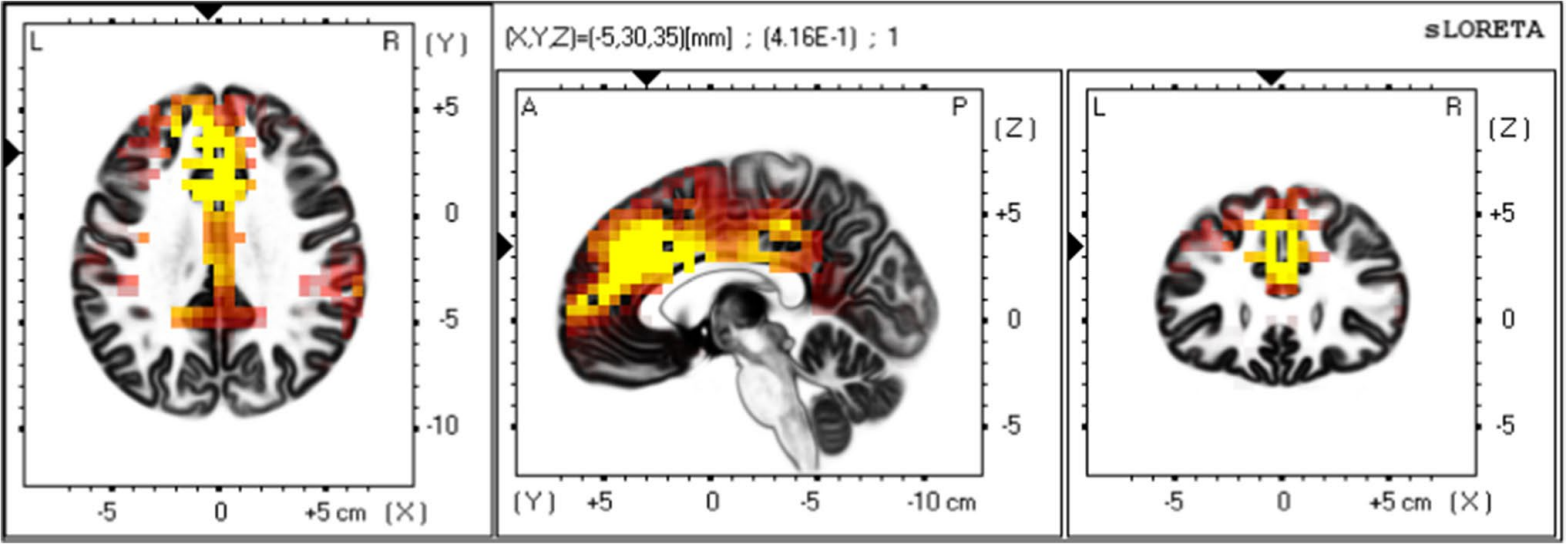
Source localization (sLORETA) results showing voxels with a significant interaction effect of condition and political orientation. Significant voxels in yellow, critical *t*-value > 3.713). Arrows at peak voxel, MNI coordinates =  − 5, 30, 35, *t*(100) = 4.159

Based on these findings, we next conducted whole-brain corrected, source localization analyses of the impact of condition, political orientation and their interaction on the paired contrast between white noise and standard tone sLORETA images, at only the P3 timeframe. Results revealed a significant interaction effect between condition and political orientation in 47 voxels in clusters in the dorsal ACC, medial PFC, left dorsolateral PFC, frontal pole, and the left parietal cortex (Fig. 4), *beta*-value threshold corrected for multiple comparisons = 0.371. The significant voxels were located in a cluster spanning Brodmann Areas 9, 32, and 24 of the dorsomedial PFC and dorsal ACC (peak voxel MNI coordinates =  − 5, 30, 35, *b* = 0.416, *p* < 0.00003.

**Fig. 5:**
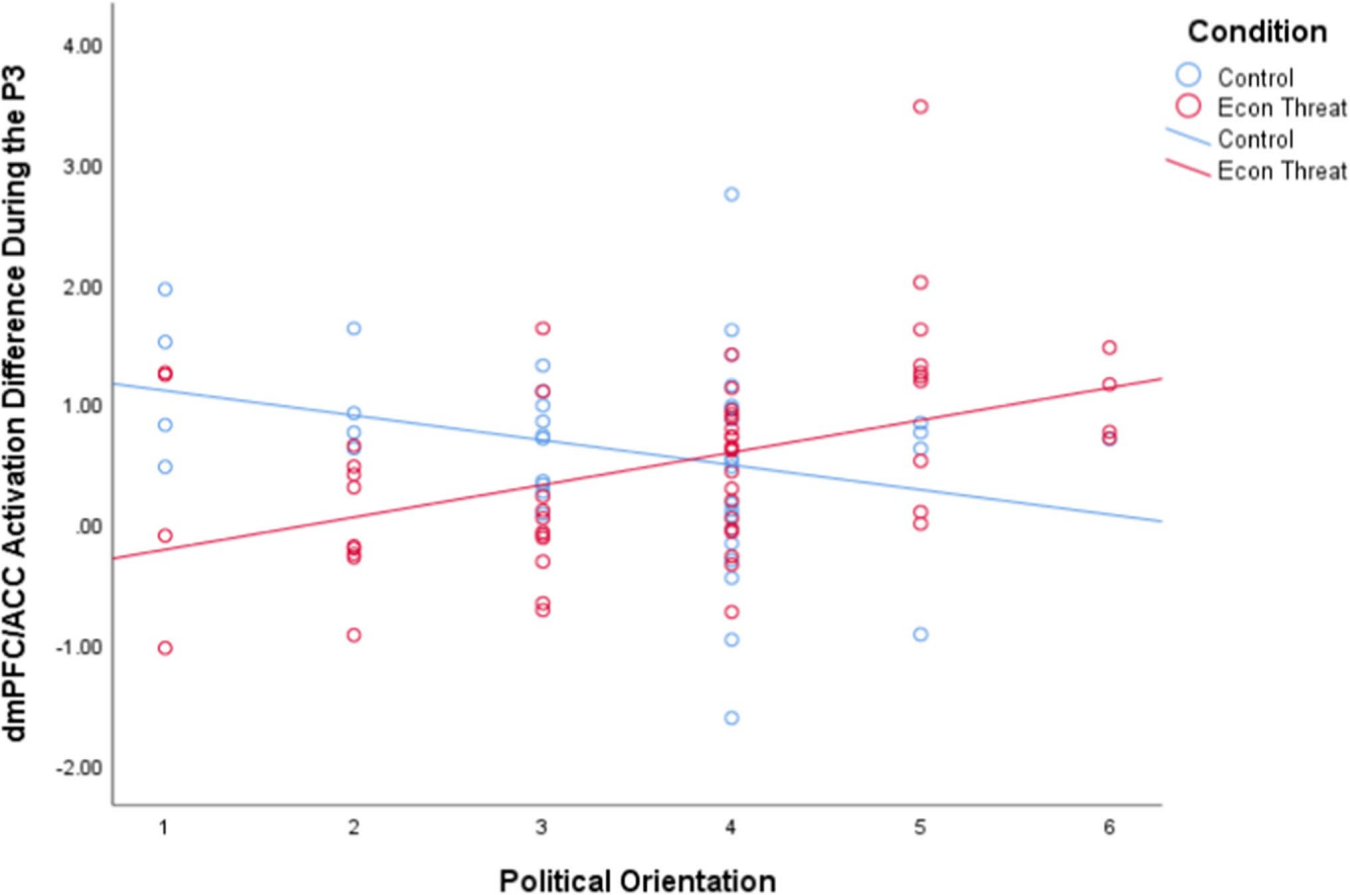
Scatterplot of the interaction between condition and political ideology on source localized activation difference in the dmPFC/ACC during the P3 component (200–400 ms) to white noise versus standard tones

To explore simple effects, we next extracted individual estimates of current density across all significant voxels in the dmPFC/dorsal ACC during the same P3 timeframe (i.e., 200–400 ms after stimulus presentation) from both white noise and standard tone sLORETA images and entered these difference scores into the same moderated multiple regression analysis. Simple slope analyses revealed that in the control condition, there was a significant negative relationship between increased conservatism and dmPFC/dorsal ACC activation during the P3 timeframe, *t*(96) =  − 2.080, *p* = 0.040. In the Economic Threat condition, there was a significant positive relationship between increased conservatism and dmPFC/dorsal ACC activation during the P3 timeframe, *t*(96) = 3.618, *p* = 0.0005. Examination of the conditional effects revealed that in the Economic Threat condition, compared to the No-Threat control condition, liberals showed significantly reduced activation in the dmPFC/dACC, *t*(96) =  − 3.547, *p* = 0.0006. Conservatives showed significantly increased dmPFC/dACC activation in the Economic Threat condition, compared to the control condition, *t*(96) = 2.411, *p* = 0.018 (Fig. 5).^2^

Overall, these results demonstrate that although liberals may be more sensitive to aversive stimuli than conservatives in neutral contexts, this relationship flips in anxiety-provoking contexts. That is, in the context of economic threat, liberals become less sensitive and conservatives become more sensitive to aversive stimuli.

### Discussion

Theory and evidence on the neuropsychology of political conservatism is divided according to two generalized views. Based on the psychological defense view, conservatism plants the individual in a stable psychological system that compensates for vulnerability or sensitivity to negative events. Based on the dispositional sensitivity view, conservatism is a cognitive consequence of dispositional sensitivity to negative stimuli and events. A seeming paradox arises. Further, research shows that conservatives appear sometimes more (Amodio et al., 2007; Kanai et al., 2011; Nash et al., 2017; Weissflog et al., 2013) and sometimes less (Dodd et al., 2012; Fodor et al., 2008; Oxley et al., 2008) sensitive to negative stimuli and events.

We reported about two studies that demonstrated that both literatures may be correct. These two studies were conducted in two different countries (New Zealand and Canada). In both studies (though more clearly in Study 2), we found that conservatism was associated with lower levels of P3 mean amplitudes to white noise stimuli after a non-threatening and neutral control condition, consistent with the idea that conservatism normally acts a psychological defense. However, after experiencing an economic threat, an experience that should compromise the psychological foundations of that protective ideology, conservatism was associated with increased P3 mean amplitudes to white noise stimuli. When conservatives face a challenge to the clarity and certainty afforded by the conservative ideology, the psychological defense falls, and conservatives shift to heightened sensitivity to negativity, threat, and/or ambiguity. In sum, the psychological defense view (e.g., motivated social cognition, Jost et al., 2003) and the dispositional sensitivity view (e.g., negativity bias, Hibbing et al., 2014) may be integrated.

Source localization results in Study 2 revealed that conservatives demonstrated heightened activation in the dorsal ACC/dorsomedial PFC, the left dorsolateral PFC, the frontal pole, and the left parietal cortex during the P3 component to white noise. This broad pattern of activation is broadly consistent with the locus coerulus-norepiniphrine (LC-NE) hypothesis of the P3 (Aston-Jones & Cohen, 2005; Nieuwenhuis et al., 2005). According to this account, the LC-NE functions to potentiate cortical representation and facilitate responding to motivationally salient stimuli. Encountering such stimuli causes a phasic increase in LC activation, prompting a burst of NE release throughout the cortex to amplify the gain of target neurons. Within this framework, the P3 reflects this distributed pattern of phasic activation and represents a cortical analogue of physiological arousal. Further, the dorsomedial PFC and the dorsal ACC are reliably associated with anxious worry (Kalisch & Gerlicher, 2014) and conflict detection (Botvinick et al., 2004; Miller and Cohen, 2001), respectively. Conservatives demonstrated heightened dmPFC and dACC activation to white noise after the economic threat, compared to the control condition. This suggests that amongst conservatives, the economic threat caused heightened negative arousal and conflict to aversive stimuli.

Our findings raise a key question, however. Does liberalism not afford psychological defense of some kind? For example, research shows that instead of a conservative shift after a threatening experience, people become more entrenched or more polarized in their preferred political orientation—i.e., liberals become more liberal and conservatives become more conservative (Bassett et al., 2015; Castano et al., 2011; Greenberg & Jonas, 2003; Toner et al., 2013). Social identity theory (Hogg, 2000, 2007) holds that uncertainty is countered by increased identification with an ingroup, and the reactive approach motivation model (McGregor et al., 2010a) holds that ideals and identities act as consistently available abstract goals to approach and quell ongoing anxieties. Presumably, these models apply to liberal ideals and identities. In the current research, liberals became less sensitive to negative stimuli after an economic threat (though evidence for this effect was not entirely consistent and was stronger in Study 2). We speculate that liberals are dispositionally more open to uncertainty, novelty and ambiguity, but respond defensively to anxiety-provoking events. In other words, conservatives may be more consistently defensive, whereas liberals are more situationally defensive. Indeed, liberals have been shown to respond defensively to threat, evidencing an apparent shift towards the psychological sanctuary of more conservative belief (Nail et al., 2009) or a “rally round the flag” effect of increased support for cultural leaders and symbols (Crawford, 2017). Of course, this liberal response may be dependent on the context, i.e., the type of threat may be important in determining whether liberals are defensive or not. Future research is needed to address these speculations.

Certain limitations in the current research afford other opportunities for future research. First, it is unclear whether the current findings generalize to other threatening experiences or different levels of economic threat. Future research could explore other anxiety-provoking events that have interacted with political orientation in past research, such as thoughts of death (Pyszczynski et al., 2006). Relatedly, future research could further support the current results and manipulate economic threat in novel ways for conceptual replication or extend this research to different intensities of threat. Second, our measure of political orientation did not consider social and economic dimensions. It may be that economic conservatives, and not social conservatives, are more impacted by economic threat. Furthermore, our measure revealed that there were fewer very conservative people than very liberal people in our sample. Future research could utilize measures capable of teasing apart these dimensions and samples that might capture the full political spectrum more evenly. Finally, our research does not clarify if the negativity bias amongst conservatives is general (all types of negative stimuli) or specific. White noise stimuli, in these studies, constitute expectancy violations or conflict, provoke arousal, and are aversive per se. Future research could manipulate these features (conflict, arousal, and negativity) to determine if conservatives are more sensitive to these types of stimuli and if this sensitivity is similarly responsive to context (i.e., threat events).

Overall, however, the current research reflects a key step in understanding the neural mechanisms in political orientation and, in particular, the link between conservatism and a negativity bias. Specifically, that link is sensitive to context (Pliskin et al., 2020). Conservatism appears to psychologically insulate from negative events and stimuli, consistent with a psychological defense view of conservatism. However, if that psychological defense is threatened, conservatism is associated with heightened vulnerability to negative events and stimuli, consistent with a dispositional sensitivity view of conservatism. This research thus helps to reconcile seemingly opposing findings and theories on the mechanisms of political orientation.

## Footnotes

1 Study 1 main effects. Results showed a marginal effect of condition, *t*(56) =  − 1.880, *p* = 0.065, and no effect of political orientation on the white noise P3 mean amplitude, *t*(56) = 1.255, *p* = 0.215. Additionally, results again showed a marginal effect of condition, *t*(56) =  − 1.698, *p* = 0.095, and no effect of political orientation on the white noise P3 mean amplitude difference score, *t*(56) = 0.917, *p* = 0.363.

2 Study 2 main effects. Results showed a significant effect of condition, *t*(96) =  − 2.875, *p* = 0.005, and a significant effect of political orientation on the white noise P3 mean amplitude, *t*(96) =  − 2.002, *p* = 0.048. Additionally, results again showed a significant effect of condition, *t*(96) =  − 2.647, *p* = 0.009, and a marginal effect of political orientation on white noise P3 mean amplitude difference score, *t*(96) =  − 1.946, *p* = 0.055. Finally, results demonstrated a significant effect of condition, *t*(96) =  − 3.922, *p* = 0.0002, and a significant effect of political orientation on dmPFC/dorsal ACC activation during the P3 timeframe, *t*(96) =  − 2.080, *p* = 0.042.
